# Irrigation water salinity structures the bacterial communities of date palm (*Phoenix dactylifera*)-associated bulk soil

**DOI:** 10.3389/fpls.2022.944637

**Published:** 2022-08-04

**Authors:** Dinesh Sanka Loganathachetti, Fardous Alhashmi, Subha Chandran, Sunil Mundra

**Affiliations:** ^1^Department of Biology, College of Science, United Arab Emirate University, Al Ain, United Arab Emirates; ^2^Khalifa Center for Genetic Engineering and Biotechnology, United Arab Emirates University, Al Ain, United Arab Emirates

**Keywords:** bulk soil, irrigation water salinity, metabarcoding, arid agroecosystem, microbial diversity, microbial communities

## Abstract

The irrigation of date palms (*Phoenix dactylifera*) with saline groundwater is routinely practiced in the agroecosystems of arid environments because of freshwater scarcity. This leads to salts deposition in topsoil layers and increases soil salinization. However, how different irrigation sources affect soil microbiota is poorly understood. Bulk soil samples were collected from date farms receiving non-saline water and saline groundwater to examine bacterial communities using metabarcoding. Overall, bacterial diversity measures (Shannon diversity index, richness, and evenness) did not vary between irrigation sources. Bacterial communities were structured based on irrigation water sources and were significantly associated with their electrical conductivity. Of 5,155 operational taxonomic units (OTUs), 21.3% were unique to soil irrigated with saline groundwater, 31.5% received non-saline water irrigation, and 47.2% were shared. The Proteobacteria abundance was higher in soil under saline groundwater irrigation while Actinobacteriota abundance was lower. A compositional shift at the genera level was also evident; the abundance of *Subgroup_10* and *Mycobacterium* was higher under saline groundwater irrigation. *Mycobacterium* was a key indicator of OTU under saline groundwater irrigation while *Solirubrobacter* was an indicator of non-saline water irrigation. Functional gene analyses showed enrichment of fatty acid, cell wall, and starch biosynthesis pathways in soil under saline groundwater irrigation. These findings provide insights into how “salinity filtering” influences bacterial communities, key taxa, and the potential metabolic function in soil under increasing irrigation water salinities, and have broad implications for arid agroecosystems.

## Introduction

Arid ecosystems are characterized by higher evapotranspiration and decrease in precipitation, which leads to the “primary salinization” of soil and a subsequent increase in groundwater salinity. As a result of low freshwater availability, saline groundwater is commonly used for irrigating the date palm (*Phoenix dactylifera*) in arid environments, resulting in ‘secondary salinization’ (Egamberdieva et al., 2010). Crop management using saline groundwater irrigation can cause insufficient percolation of water and accumulation of salts in the top veneer of soil, which, in turn, affects agricultural productivity (Chen et al., 2019). Saline water irrigation is known to induce perturbations in soil pH and electrical conductivity (EC), mediated by changes in the composition of cations (sodium, calcium, and magnesium) and anions (chloride and carbonate; Guo et al., 2020b), which, in turn, influence turnover of available nutrients (Yuan et al., 2018). Apart from water and soil chemistry-related changes, salinization also affects soil properties like flocculation and dispersion which are critical for maintaining soil structure and facilitating water movement (Rengasamy, 2018). These adverse edaphic changes associated with saline water irrigation can further affect the belowground microbiota.

Soil salinity is an important determinant of bacterial richness (Zhang et al., 2019; Li et al., 2021b) and Shannon diversity index (Guo et al., 2021; Yu et al., 2021; Nan et al., 2022) in arid ecosystems. Apart from soil salinity, irrigation water salinity also affect the Shannon diversity index, both negatively (Zheng et al., 2017; Chen et al., 2019; Hou et al., 2021) and positively (Chen et al., 2017) in arid soil. Soil salinity is also reported to structure bacterial communities (O’Brien et al., 2019; Rath et al., 2019; Guo et al., 2021; Li et al., 2021a; Nan et al., 2022), with soil pH and EC being major predictors of bacterial communities (O’Brien et al., 2019; Rath et al., 2019; Nan et al., 2022) in arid ecosystems. Despite gaining limited knowledge on the impact of groundwater irrigation-induced salinity on bacterial communities, the responsible structuring factor has not been identified yet (Chen et al., 2017). Moreover, one study found no changes in arid agricultural soil microbial communities following saline water irrigation (Ibekwe et al., 2017). Thus, inconsistencies with regard to the impact of irrigation water on soil bacterial diversity and communities in arid agroecosystems require further attention to better understand community dynamics.

The resiliency of soil-inhabiting bacteria against increasing salinity is dependent on the filtration of specific bacterial taxa and capable of withstanding the stress. Based on previous studies, soil salinity increase is known to correlate with an increase in abundance of Proteobacteria (Nan et al., 2022) and its classes, Gamma and Alphaproteobacteria (Zhang et al., 2019), and a reduction in Actinobacteriota, Chloroflexi, Acidobacteria, and Planctomycetes (Li et al., 2021a). In cotton field soil, Chen et al. (2019) found a reduction in the abundance of Actinobacteriota, Gemmatimonadetes, and Acidobacteria after irrigation with saline groundwater. Another study in which saline groundwater with different saline ranges was used for irrigating cotton field soil, showed an increase in Proteobacteria and Actinobacteriota and a decrease in Planctomycetes and Bacteroidetes (Chen et al., 2017). Soil salinity is shown to increase the abundance of *Halobacteria* and *Nitriliruptoria* genera in maize (Li et al., 2021a) and *Rhodanobacter, Acidobacterium, Candidatus Nitrosotalea,* and *Candidatus Koribacter* in barley field soil (Li et al., 2021b). These bacterial taxa present in arid soils of agroecosystems possibly withstand salinity stress by producing spores, extracellular polysaccharides (EPS), antioxidant enzymes, and osmolytes that possibly confer salinity tolerance in arid soil (Alsharif et al., 2020). In addition, bacteria play a vital role in biogeochemical cycling in terrestrial environments including hyper-arid to arid habitats. Previous studies indicate that the abundance of genes specific for ammonia oxidation (Guo et al., 2020a; Khan and Khan, 2020), nitrogen fixation (Ren et al., 2018; Khan and Khan, 2020), denitrification (Ren et al., 2018; Guo et al., 2021), and sulfate production (Guo et al., 2021) are known to modulate with respect to increase in soil salinization. Increasing soil salinity also stimulates nitrous oxide (greenhouse gas) in desert soil (Zhang et al., 2016), which can inhibit the growth of bacteria involved in nitrogen cycling (Li et al., 2021a). Thus, it is evident that soil salinization-induced changes in the selection of microbial taxa and their functionality can potentially impact the functioning of global ecosystems.

Current knowledge of saline groundwater irrigation-induced secondary salinization on bacterial communities and diversity remains inconsistent, possibly because of limited sampling sites and ranges of salinity. Thus, a comprehensive study that includes higher salinity ranges of irrigation water that prevail in the agroecosystem of arid environments is critical. We aimed to assess the impact of irrigation sources (non-saline water and saline groundwater) on date palm-associated bulk soil bacterial diversity, communities, structuring factors, and potential functional pathways. We also investigated key indicator taxa that responded to changes in irrigation water sources. We hypothesized that ‘salinity filtering’ is the key factor responsible for structuring bacterial communities and their compositional patterns.

## Materials and methods

### Study site description and sample collection

The study sites were located in the oasis agroecosystem of Al Ain (Abu Dhabi Emirates, United Arab Emirates). The climate of the study sites is classified as “Bwh” (tropical and subtropical desert climate) according to the Köppen climate classification. The mean annual temperature (MAT; 25.3–27.8°C)^1^ and mean annual rainfall (MAP; 75–109 mm) (see Footnote 1) of sampling sites showed characteristics of dryland ecosystems (Supplementary Table S1). Sampling was performed in 14 study sites (i.e., date palm farms) and bulk soil (hereafter referred to as “soil”) was collected in March 2020. Seven of the sites were irrigated with non-saline freshwater (hereafter referred to as “water”) and seven with saline groundwater. The electrical conductivity was 0.65 ± 0.20 ds m^−1^ (pH range 6.96–7.99) and 11.55 ± 1.7 ds m^−1^ (pH range 7.33–7.72) for non-saline water and saline groundwater, respectively. Three replicated soil samples were collected from each study site approximately 50 m away from each other, at a depth of 20–30 cm. In total, 42 soil samples (seven farms × two irrigation sources × three replicates) were collected for both chemical and molecular analyses. The irrigation water was also collected from each farm for chemistry analyses. All the samples were transported to the lab in cool conditions and soil samples for molecular analyses were stored at −20°C until DNA isolation.

### Soil and water chemistry analyses

Soil samples were passed through a 2 mm sieve to remove plant debris and pulverized using a mixer mill. One gram of fine soil was mixed thoroughly with 9 ml of milliQ water and homogenized for 1 h at 200 rpm. This soil-water mixture was passed through Whatman filter paper and the filtrate was used for measuring soil chemistry (EC and pH), while water chemistry (pH and EC) was determined by directly analyzing the water samples. Soil organic matter (OM) of the samples was measured based on loss on ignition method (Nelson and Sommers, 1996). In brief, 5 g of air-dried soil samples were kept at 360°C for 4 h, and loss of mass after incubation was used to calculate the soil OM.

### Soil DNA isolation and illumina sequencing

Deoxyribonucleic acid (DNA) isolation was performed using the E.Z.N.A soil DNA kit according to the manufacturer’s protocol. The 341F (CCTACGGGNGGCWGCAG) and 805R (GACTACHVGGGTATCTAATCC; Herlemann et al., 2011) primer combination was used to amplify the V3–V4 region of 16S rRNA. A 50 μl PCR reaction consisting of forward and reverse primers (1 μM each), 250 μM dTNPs (0.5 μM of each), 0.02 U Phusion High-Fidelity DNA polymerase (Finnzymes, Finland), 0.3 mg/ml Bovine Serum Albumin (BSA), and 5 × Phusion HF buffer containing 1.5 mM MgCl_2_ was initiated. The PCR conditions consisted of initial denaturation at 95°C for 5 min, 25 repeating cycles of denaturation (95°C for 40 s), annealing (55°C for 30 s), and extension (72°C for 1 min), and a final extension step (72°C for 7 min). A DNA Normalization Kit (Charm Biotech, United States of America) was used for the purification and normalization of PCR amplicons. MiSeq (2×300 bp; paired-end) sequencing was performed at IMR Lab, Halifax, Canada^2^ following the standard Illumina protocol. The demultiplexed raw sequence data were uploaded to the Zenodo repository.^3^

### Bioinformatics analyses

A total of 907,507 raw sequence reads were analyzed using the Divisive Amplicon Denoising Algorithm 2 v1.12 (*DADA2*) R package (Callahan et al., 2016). The forward and reverse primer sequences present in the raw sequence data files (R1 and R2) were trimmed using the rbind function of the *SparkR* package. After primer removal, the sequences were processed for quality filtering (max *N* = 0, truncQ = 2, maxEE = 2) and trimming (> 275 bp for forward, > 225 bp for reverse reads) using the filterAndTrim function. The trimmed reads were subsequently processed for error model generation (learnErrors), denoising (dada), merging (mergePairs), amplicon sequence variant (ASV) inference, and chimera removal (removeBimeraDenovo) using the respective functions of *DADA2*. Additional clustering of ASVs to operational taxonomic units (OTUs) at 97% sequence similarity was performed using *vsearch* v2.15 (Rognes et al., 2016), resulting in 8,429 OTUs (541,004 reads). After clustering, singleton and chimera screening (1,245 OTUs: 12,523 reads) were carried out using *vsearch,* resulting in 7,184 OTUs (528,481 reads). The taxonomic assignment of non-chimeric OTUs (7,184 OTUs: 528,481 reads) was carried out using the Silva database v138.1 (downloaded from https://zenodo.org/record/4587955) using the assignTaxonomy function (Quast et al., 2012) of *DADA2* based on the naïve Bayesian classifier with minBoot = 80. In total, 7,184 OTUs, 35 archaeal (185 reads), seven chloroplast (3,861 reads), four mitochondrial (346 reads), one non-bacterial (two reads), one unclassified_kingdom level OTU (five reads), and 1,980 OTUs with reads < 5 (5,702 reads) were manually removed from the OTU table. The final OTU table consisted of 5,155 OTUs (507,111 reads) from 42 samples (read range: 1,770–47,721). The OTU table was normalized based on a sample with the lowest number of sequences (1,770) using the rrarefy function of the *vegan* R package (Oksanen et al., 2020) before α- and β-diversity analyses. The OTUs were classified as abundant (> 1%), moderate (0.1–1%), and rare (< 0.1%) taxa based on the % occurrences defined previously (Dai et al., 2016). Functional prediction of OTUs that passed the Nearest Sequenced Taxon Index (NSTI; < 2) was carried out using the Phylogenetic Investigation of Communities by Reconstruction of Unobserved States (PICRUSt2; Langille et al., 2013). Normalized OTU counts of samples were used for the KEGG orthologs (KO) assignment and KO assignments were used to predict the MetaCyc pathway abundance of bacteria in soil under non-saline water and saline groundwater irrigation using PICRUSt2.

### Statistical analyses

All statistical analyses in this study were performed using R v4.0.3 (R Core Team, 2020). Prior to analyses, the OTU count data of samples were arcsine-transformed to increase the homogeneity of variance. The water (pH and EC) and soil (pH, EC, and soil OM) chemistry values were standardized to a 0–1 scale using Z transformation. Analyses of variance (ANOVA) followed by Tukey’s HSD *post hoc* test were performed using the *agricolae* package to test for differences in the soil chemistry (pH, EC, and OM), water chemistry (pH and EC), and bacterial diversity (bacterial richness, Shannon diversity index, and evenness) between irrigation water sources (non-saline water and saline groundwater irrigation). Bacterial OTUs with a 0.02% relative abundance in at least 80% of samples (28 of 35 samples) were defined as core taxa (Gschwend et al., 2022) using the microbiomeAnalyst web platform (Dhariwal et al., 2017). The indicator OTU analysis was performed using the multiplatt function of the *indicspecies* R package and an indVal (> 0.5) with *p* < 0.05 was used to predict indicator OTU in soil under non-saline water and saline groundwater irrigation. To assess the effect of environmental variables on bacterial community structuring patterns between irrigation water sources (non-saline water and saline groundwater irrigation), two-dimensional non-metric multidimensional scaling (NMDS) analyses based on Bray–Curtis dissimilarities were carried out using the metaMDS function of the *vegan* package (Oksanen et al., 2020). NMDS analyses were performed using the following settings: dimensions (*k*) = 2, maximum iterations = 1,000, initial configurations = 100, minimum stress improvement in each iteration cycle = 10^−5^ to find a stable solution with low stress values. The vectors associated with environmental factors (*p* < 0.05) and the centroids that represented irrigation water sources (non-saline vs. saline groundwater irrigation) were fitted to an NMDS ordination plot using the envfit function and 95% confidence intervals (CI) of the plots were generated using the ordiellipse function of the *vegan* package (Oksanen et al., 2020). Permutational analysis of variance (PERMANOVA) was carried out using the adonis function of the *vegan* package (Oksanen et al., 2020) to test significant differences of bacterial communities between irrigation water sources (non-saline vs. saline groundwater irrigation), while pseudo-F statistics was computed by performing 9,999 permutations of Bray-Curtis dissimilarities. A forward selection procedure was used to optimize the final model for PERMANOVA analyses (Blanchet et al., 2008). Initially, single-factor models were performed and factors were ranked based on their significance (*p* < 0.05) and R^2^ values in the final model. In addition, the homogeneity of bacterial communities in soil under non-saline and saline groundwater irrigation and their statistical significance were analyzed using PERMDISP function of Primer6 software by computing 999 permutations of Bray-Curtis dissimilarities.

## Results

### The impact of irrigation water on chemical and microbial analyses

The level of EC was significantly higher in saline groundwater (11.55 ± 1.7 ds m^−1^) than non-saline water (0.65 ± 0.2 ds m^−1^). Contrarily, pH of saline groundwater (7.54 ± 0.03) was significantly lower than non-saline water (7.73 ± 0.07; Supplementary Figures S1A,B). No significant changes were found in soil pH and EC between irrigation sources; however, the soil OM was significantly lower in soil under saline groundwater irrigation (2.56 ± 0.26) than in non-saline water (4.21 ± 0.35; Supplementary Figure S1C).

**Figure 1:**
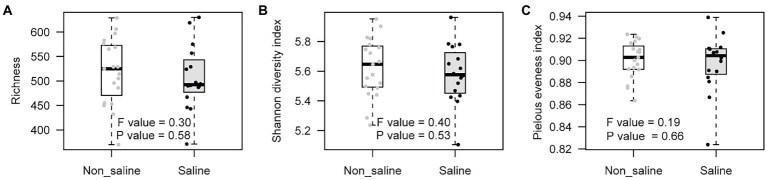
Box-plot of bacterial diversity in soil under non-saline water and saline groundwater irrigation. **(A)** Richness, **(B)** Shannon diversity index, and **(C)** Pielou’s evenness index.

**Figure 2:**
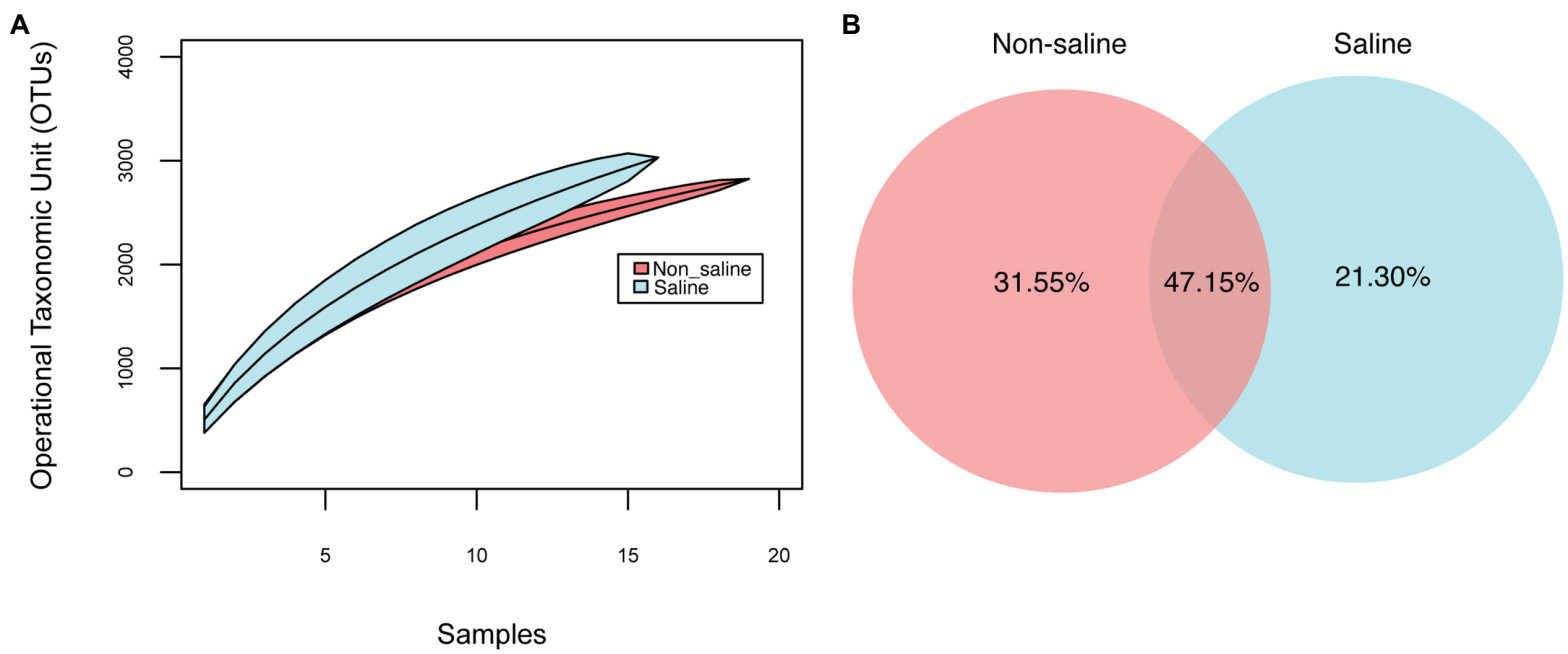
Species accumulation curves, unique and shared bacterial OTU analyses. **(A)** Operational taxonomic unit (OTU) accumulation curves at 97% sequence similarity and **(B)** shared and unique OTUs of date palm-associated soil between irrigation sources (non-saline vs. saline groundwater irrigation). The unique and shared OTUs are expressed as percentages of total OTUs (5,155).

**Figure 3:**
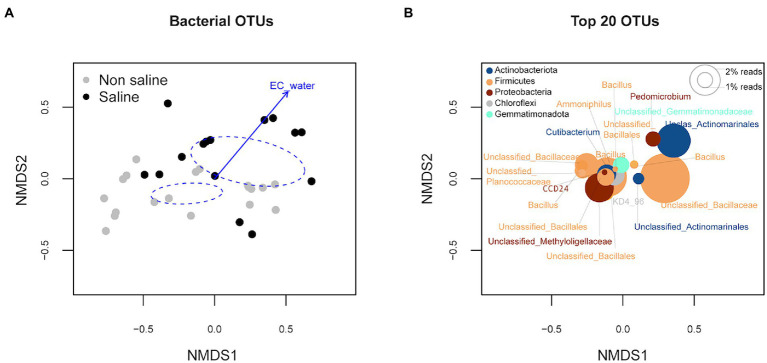
Multivariate non-metric multidimensional scaling (NMDS) ordination analysis of bacterial communities in soil under non-saline water and saline groundwater irrigation. **(A)** The ordination plot was generated based on the operational taxonomic unit (OTU) abundance of soil under non-saline and saline groundwater irrigation. The colors are coded according to **(A)** the irrigation water source (non-saline vs. saline groundwater irrigation) and **(B)** bacterial phyla. The 95% ellipse represents the confidence interval for the tested factor variable (i.e., irrigation water source) and direction and length point increased influence of the significant variable (*p* < 0.05) on the bacterial communities of samples in the pointing direction of ordination configuration. **(B)** Species plots of the top 20 bacterial taxa based on total OTU composition. The size of the circles indicates the relative abundance of the OTUs.

Soil bacterial diversity (richness, Shannon diversity index, and evenness) parameters were not significantly different under non-saline water and saline groundwater irrigation (Figure 1). Rarefaction curves of soil bacteria did not reach a plateau for both types of soil samples under non-saline water and saline groundwater irrigation (Figure 2A). Of 5,155 OTUs, 21.3% were only detected in soil under saline groundwater compared to 31.5% detected under non-saline water irrigation, and 47.15% were shared between both soils (Figure 2B). Soil bacterial communities differed significantly between irrigation water sources (non-saline vs. saline groundwater irrigation) based on multivariate (PERMANOVA and NMDS ordination) analyses. Two distinct clusters representing non-saline water and saline groundwater irrigation were observed in NMDS ordination space (*R*^2^ = 0.1503, *p*-value = 0.013; Figures 3A,B). The final model of PERMANOVA analyses obtained using the forward selection procedure showed that out of five tested factors (soil pH, soil EC, water pH, water EC, and soil OM), only irrigation water EC (*R*^2^ = 0.1825, *p*-value = 0.043) significantly affected bacterial community structural patterns. The bacterial communities of soil under saline groundwater irrigation were homogeneous (*F*-value = 57.167; *p*-value = 0.014), whereas non-saline water irrigation sites showed heterogeneous distribution (*F*-value: 7.335; *p*-value = 0.134).

### Irrigation water impact on bacterial composition and potential functions

**Figure 4:**
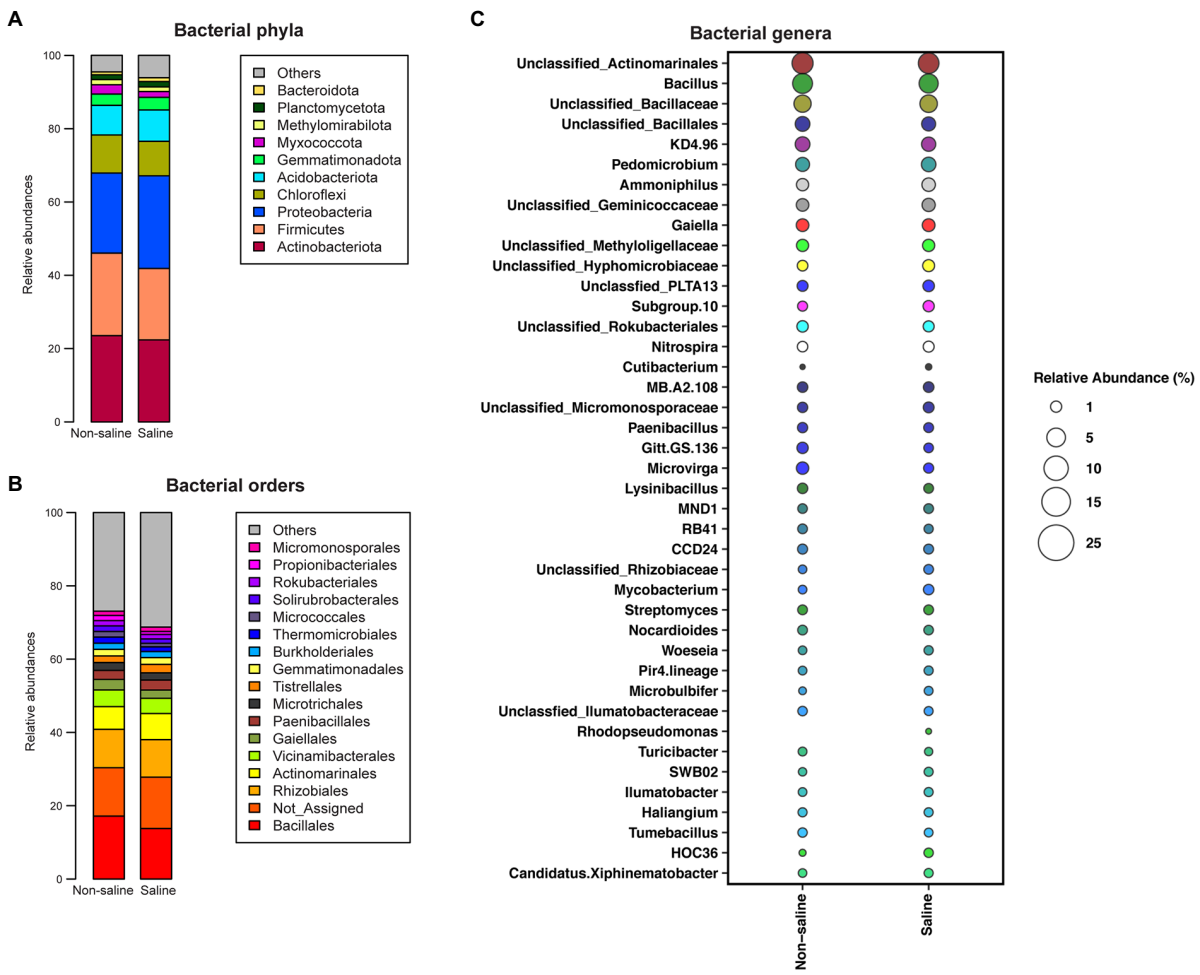
The relative abundance of the top bacterial taxa in soil. The relative abundance of bacteria in the soil at the **(A)** phylum, **(B)** order, and **(C)** genus levels.

Actinobacteriota (24.4%), Firmicutes (23.2%), Proteobacteria (22.8%), Chloroflexi (11%), Acidobacteriota (9.1%), Gemmatimonadota (3.5%), Methylomirabilota (1.48%), and Planctomycetota (1.7%) were the abundant phyla (> 1% abundance) in soil under non-saline water irrigation, while Proteobacteria (27.9%), Actinobacteriota (23.01%), Firmicutes (21.9%), Chloroflexi (10%), Acidobacteriota (9.4%), Gemmatimonadota (3.7%), Planctomycetota (2%), and Methylomirabilota (1.3%) were abundant (> 1% abundance) phyla in soil under saline groundwater irrigation (Figure 4A; Supplementary Table S2). Of these phyla, the relative abundances of Chloroflexi, Acidobacteriota, Gemmatimonadota, Methylomirabilota, and Planctomycetota were unchanged between irrigation water sources. Bacilli (17.7, 21.9%) was enriched in soil under non-saline water irrigation, while the Proteobacterial classes, Gamma (8, 5.5%) and Alphaproteobacteria (17.6, 16%), were enriched in soil under saline water irrigation. The soil samples consisted of the following orders, Bacillales, Rhizobiales, Actinomarinales, Vicinamibacterales, Paenibacillales, Tistrellales, Gaiellales, Microtrichales, Gemmatimonadales, Burkholderiales, Thermomicrobiales, and Rokubacteriales as top taxa with varied abundances under non-saline water and saline groundwater irrigation (Figure 4B; Supplementary Table S2). *Bacillus*, *Pedomicrobium,* and *Gaiella* were the top genera detected in soil under both irrigation water sources (Figure 4C; Table 1). The *Microvirga, Ammoniphilus, Nitrospira*, and *Lysinibacillus* genera were highly dominant in soil under non-saline water irrigation. Similarly, *Subgroup_10, Nitrospira*, and *Mycobacterium* were the top genera in soil collected from saline groundwater irrigation.

**Table 1:** Taxonomic affinity, read abundance, and occurrence of the 20 most abundant operational taxonomic units (OTUs) detected in soil under non-saline water and saline groundwater irrigation.

OTU ID	Genus (Phylum)^**^	Overall		Non-saline		Saline	
		Reads (%)	Occurrence (%)^*^	Read (%)	Occurrence (%)^#^	Reads (%)	Occurrence (%)^$^
OTU3^†^	Actinomarinales_unclassified (A)	2.54	94.29	2.67	89.47	2.42	100
OTU5	Ammoniphilus (F)	1.91	100	1.39	100	2.55	100
OTU8	Bacillales_unclassified (F)	1.42	100	1.75	100	1.24	100
OTU1	Bacillaceae_unclassified (F)	1.15	85.71	0.64	89.47	1.72	81.25
OTU1633^†^	Bacillus (F)	1.16	100	0.70	100	0.46	100
OTU14^†^	Pedomicrobium (P)	1.02	97.14	1	100	1.08	93.75
OTU15	Methyloligellaceae_unclassified (P)	0.95	100	0.93	100	1.01	100
OTU37	Bacillus (F)	0.96	100	0.45	100	0.51	100
OTU6	Bacillaceae_unclassified (F)	0.89	100	1.03	100	0.88	100
OTU66	Actinomarinales_unclasssified (A)	0.93	97.14	0.56	100	0.37	93.75
OTU16	Bacillus (F)	0.86	100	0.83	100	0.92	100
OTU17	PLTA13 (P)	0.83	97.14	0.74	100	0.92	93.75
OTU9	KD4-96_unclassified (C)	0.78	97.14	0.84	100	0.79	93.75
OTU7	Bacillus (F)	0.78	74.29	0.10	68.42	1.38	81.25
OTU24	Gemmatimonadaceae_unclassified(G)	0.71	97.14	0.36	100	0.35	93.75
OTU27	Bacillus (F)	0.60	100	0.29	100	0.31	100
OTU25	MB-A2-108_unclassified (A)	0.45	97.14	0.24	100	0.21	93.75
OTU10	Planococcaceae_unclassified (F)	0.53	80	0.24	73.68	0.89	87.50
OTU11	Bacillaceae_unclassified (F)	0.57	100	0.46	100	0.69	100
OTU51	CCD24_unclassified (P)	0.54	97.14	0.28	100	0.26	93.75

Total reads (%) and occurrences among samples were calculated for the overall database, non-saline sample, and saline sample subset.

** The abbreviations (A), (P), (F), and (G) represent the bacterial phyla, Actinobacteriota, Proteobacteria, Firmicutes, and Gemmatimonadota, respectively.

† indicates core taxa with 0.02% reads across at least 28 samples (80%).

* Occurrence (%) calculated from all 35 samples.

# Occurrence (%) calculated from the 19 non-saline samples.

$ Occurrence (%) calculated from the 16 saline samples.

**Figure 5:**
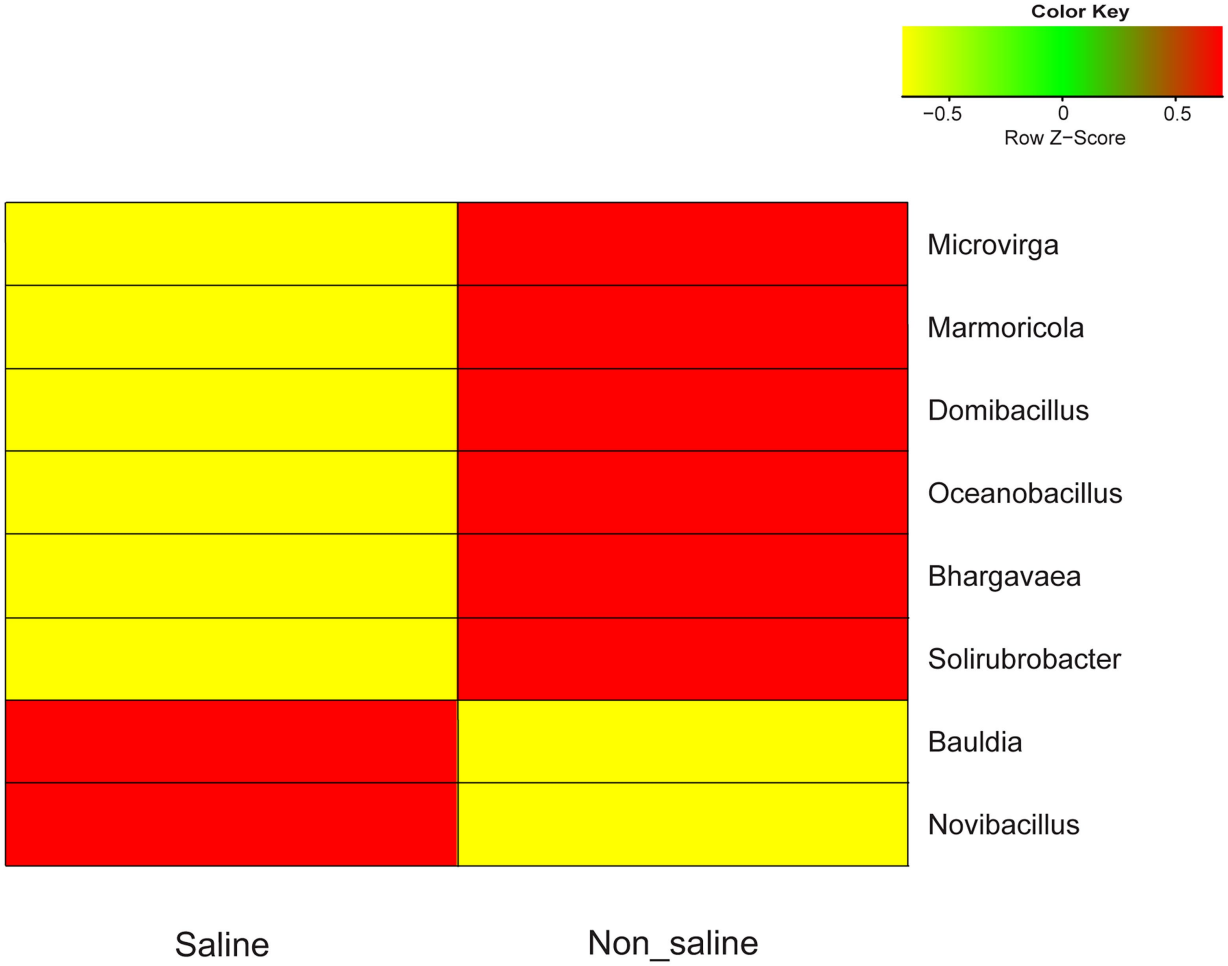
Heatmap of bacterial OTU abundances in soil under non-saline water and saline groundwater irrigation. The hierarchical clustering of significant operational taxonomic units (OTUs; *p* < 0.05) of soil is shown under saline vs. non-saline water irrigation. The color key of the legend indicates the median-centered *Z*-score values, which were calculated after normalizing the relative abundance values of selected genera.

The most common OTUs belonging to the *Microvirga*, *Marmoricola*, *Domibacillus*, *Oceanobacillus*, *Bhargavaea*, and *Solirubrobacter* genera were significantly abundant in soil under non-saline water irrigation, while *Novibacillus* and *Bauldia* were more common under saline groundwater irrigation (Figure 5). The proportions of these significantly differing taxa detected in soil under both types of irrigation water sources were at a moderate level (< 0.1 to 1% abundance). The core taxa detected in the study were Actinomarinales_unclassified, *Bacillus*, and *Pedomicrobium* in soil under both types of irrigation water sources (Table 1). Several indicator OTUs specific for the soil under non-saline water (6 OTUs) and saline groundwater (two OTUs) irrigation were detected in this study. The topmost indicator OTUs with the highest indVal in soil were *Solirubrobacter* and *Sorangium* under non-saline water irrigation and *Mycobacterium* and *Steroidobacter* under saline groundwater irrigation (Supplementary Table S3).

**Figure 6:**
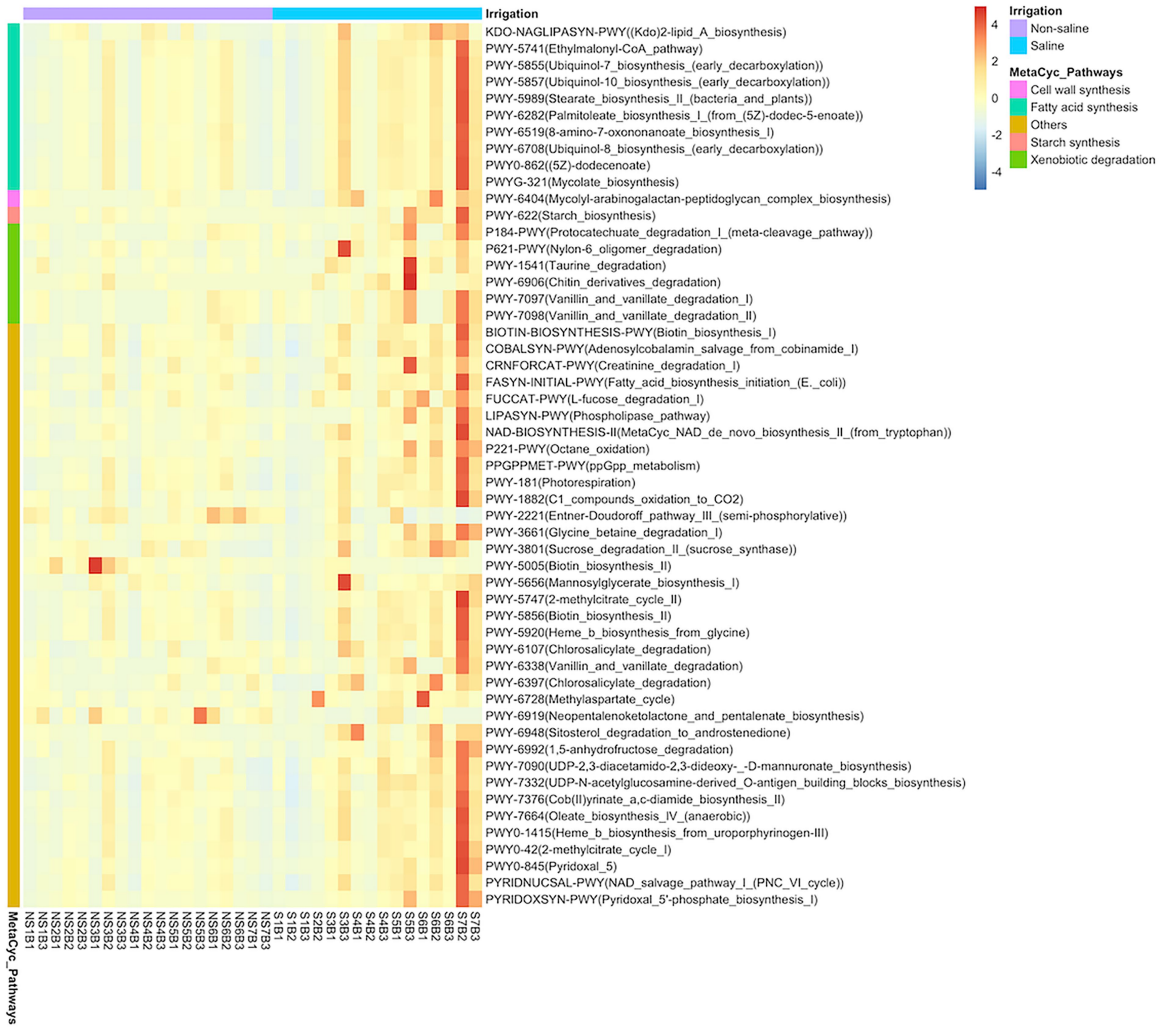
Heatmap analysis of predicted MetaCyc pathway abundances of bacterial OTUs. The heatmap shows significant predicted MetaCyc pathways (*p* < 0.05) of bacteria in soil under different irrigation water sources (non-saline water vs. saline groundwater) using PICRUSt2 software. The MetaCyc pathways are grouped into categories and color key indicates the relative abundance of pathways on a scale of −4 to 4.

Of the 248 pathways predicted based on bacterial OTU composition in this study, 54 differed significantly (*p* < 0.05) in soil under both non-saline water and saline groundwater irrigation (Figure 6). Among the significantly varying pathways, genes related to fatty acid metabolism (Stearate biosynthesis II, Palmitoleate biosynthesis I from 5Z-dodec-5-enoate, Ubiquinol-8 biosynthesis, and Oleate biosynthesis IV), cell wall (Mycolyl-arabinogalactan-peptidoglycan biosynthesis), and starch biosynthesis pathways were enriched in soil under saline groundwater irrigation.

## Discussion

No changes in soil bacterial diversity were found between irrigation sources, however, a unique set of OTUs was present under saline groundwater irrigation. These results show that irrigation water alters bacterial community composition and structural patterns are related to water EC. Moreover, the abundance of the Subgroup_10 genera, *Mycobacterium* and *Steroidobacter*, and the expression of genes associated with fatty acid and starch biosynthesis pathways were higher under saline groundwater irrigation, indicating that they use putative salinity tolerant mechanisms in arid agroecosystems. In addition to salinity, the selection pressure exerted by roots (i.e., root exudates and rhizodeposits) may also play a role. This could differ by salinity (Futamata et al., 1998) and possibly extend beyond the rhizosphere, thus allowing the growth of certain bacteria in soil (Bakker et al., 2015; Moroenyane et al., 2018).

### Saline groundwater irrigation does not impact diversity but alter compositional patterns

The soil bacterial diversity (richness, Shannon diversity, and evenness) did not vary significantly between the two irrigation sources, but the overall OTU numbers were low in soil under saline groundwater irrigation. A similar decrease in bacterial OTU numbers because of salinity has been observed in a desert ecosystem (Li et al., 2021a). We found that bacterial communities in soil under two irrigation sources were distinct and that salinity (i.e., irrigation water EC) was the major structuring factor of the communities. While 21.3% OTUs were unique in soil under saline groundwater irrigation, 31.5% were unique under non-saline water irrigation. This structuring of bacterial communities from saline groundwater possibly occurs transiently in the soil through a deterministic ‘salinity filtering’ process, wherein salt-tolerant species may replace less salt-tolerant members through species sorting. The higher salinity of irrigation water may be attributed to higher sodium and chloride concentrations (Egamberdieva et al., 2010; Khan et al., 2019), which can cause an osmotic imbalance in the surrounding soil environment of bacteria. Bacteria may become dormant or lyse due to osmotic stress-induced plasmolysis depending on their salinity tolerance capacity. Moreover, the bacterial communities of soil samples under saline groundwater irrigation were homogeneous across sites, which shows that the salinity filtering is consistent across sites in structuring bacterial communities in this study. In addition, salinity range of irrigation water may play an important role in structuring bacterial communities in agroecosystems since salinity is reported to structure bacterial communities in a concentration-dependent manner (0–22 mg NaCl g^−1^ of soil) based on a microcosm-based study (Rath et al., 2017). Irrigation water salinity range of 1.09–6.12 dS m^−1^ showed salinity-dependent distinct bacterial community structuring in cotton field soil (Chen et al., 2017). In contrast, a study on soil from a spinach field with a salinity range of 0.85–15 dS m^−1^ did not have an effect on bacterial communities (Ibekwe et al., 2017). These studies did not represent wider salinity ranges of irrigation water and account for geographical variability (Chen et al., 2017; Ibekwe et al., 2017) in arid agroecosystems yielding inconsistent results. These inconsistencies may be due to the filtration effect or adaptive capabilities of bacteria that caused changes, or the lack of changes, in soil bacterial communities. However, the results of the current study show a consistent bacterial community structuring based on irrigation water sources despite covering multiple geographical locations and a wide range of salinities (0.33–28 ds m^−1^), which indicates that our observation also accounted for patchiness in bacterial communities resulting from spatial variability. Though we did not evaluate the role of geo-climatic factors (i.e., MAT and MAP) on bacterial communities, they may also act as indirect determinant(s) of soil communities across sites.

### Saline groundwater irrigation impacts bacterial composition and their potential pathways

The higher abundance of Proteobacteria and its classes Gamma and Alphaproteobacteria in soil under saline groundwater irrigation found in this study correlate with previous studies from arid and saline environments (Chen et al., 2017, 2019; Nan et al., 2022). We found higher EC and lower OM in soil under saline groundwater irrigation and several Proteobacteria are shown to be capable of growing under such nutrient-limited and extreme environmental conditions (Chen et al., 2019; Zhang et al., 2019). The other dominant phyla, Firmicutes (*Bacillus*, *Lysinibacillus, Domibacillus,* and *Oceanibacillus*) and Actinobacteriota (*Ammoniphilus* and *Mycobacterium*), capable of performing organic matter decomposition under salinity stress, were enriched in soil and may explain the decrease in soil OM under saline groundwater irrigation observed in this study.

*Mycobacterium* sp. are well adapted to high salinity (Asmar et al., 2016) and promote plant growth (Karmakar et al., 2021) in such conditions. *Mycobacterium* was also an indicator taxon under saline groundwater irrigation, showing an increase in arabinogalactan-peptidoglycan (cell wall) synthesis that may serve as a potential mechanism for tolerating salinity stress. Subgroup_10 genus (Acidobacteriota) abundance was higher in soil under saline water irrigation, whose members are reported to accumulate starch (Kristensen et al., 2021) and starch accumulation was reported to provide osmoprotectant effect for bacteria against salinity stress (Qiao et al., 2018). The increased abundance of putative genes related to starch biosynthesis pathways under saline groundwater irrigation indicates a possible salinity tolerance mechanism adapted by Subgroup_10 members. Saline groundwater irrigation increased the abundance of *Microvirga* (Alphaproteobacteria), a free-living, nitrogen-fixing, and nitrate-reducing bacterium isolated from desert soil (Amin et al., 2016), suggesting that it may function as a nitrifier despite salinity-stress. Similarly, *Ammoniphilus,* an ammonia-oxidizing bacterium (Zaitsev et al., 1998), was more abundant in soil under saline groundwater irrigation.

Members of core taxa such as those belonging to order Bacillales (*Bacillus*, *Lysinibacillus*, *Domibacillus*, and *Oceanobacillus*) produce endospores under hot and saline conditions, secreting extracellular polymeric substances (EPS) and forming a biofilm to enhance soil aggregation (Marvasi et al., 2010). Another core taxon, *Pedomicrobium*, a biofilm-dwelling iron-oxidizing bacterium (Cox and Sly, 1997), was enriched across samples, indicating that it plays an iron oxidation role in soil possibly by attaching to biofilms produced by Bacillales members. Several plant growth-promoting rhizobacteria (PGPR) members were detected under saline groundwater (*Novibacillus*; Mandic-Mulec et al., 2015; Martínez and Dussán, 2018; Mukhtar et al., 2021) and non-saline water (*Bacillus*, *Lysinibacillus*, *Domibacillus*, *Oceanobacillus*, and *Marmoricola*) irrigation, indicating that soil may be serving as a base for bacterial recruitment to the rhizosphere of date palms.

## Conclusion

Saline groundwater did not alter bacterial diversity but did impact a small percentage of the total OTUs selected from soil samples, potentially because of osmotic stress. Further, our findings indicate that saline groundwater-mediated “salinity filtering” may distinctly alter bacterial communities compared to non-saline water irrigation in soil. The filtration of salinity-tolerant bacterial taxa (*Mycobacterium* and *Subgroup_10*) and their potential functions related to fatty acid and starch synthesis in soil under saline groundwater irrigation indicate possible bacterial adaptation mechanisms in arid agroecosystems. In addition, the presence of various PGPR bacteria (*Bacillus*, *Lysinibacillus*, *Domibacillus*, and *Novibacillus*) also occurs in soil under saline groundwater irrigation points the bulk soil role as “seed source” for potential recruitment by date palm roots. We have shown that date palm-associated soil bacterial communities show strong and consistent responses under saline groundwater irrigation despite a higher range of salinities and geographical distribution. Overall, the application of saline groundwater for irrigation alters bacterial communities of bulk soil by filtering in salt-tolerant bacteria that adapts to salinity stress by enriching specific pathways related to fatty acid and starch. Our findings provided deeper insights with regard to irrigation water salinity effects on bulk soil-associated bacterial communities of date palm, which is useful in strategizing irrigation and has broader implications in arid agroecosystems.

## Data availability statement

The datasets presented in this study can be found in online repositories. The names of the repository/repositories and accession number(s) can be found at: https://doi.org/10.5281/zenodo.6371857, zenodo.6371857.

## Author contributions

DL: performed bioinformatic and statistical analyses and drafted the article. FA and SC: performed sampling and lab work. SM: conceived the project idea, performed fieldwork, and funding acquisition. All authors contributed to the article and approved the submitted version.

## Funding

This research work is supported by a Start-up Research Grant (G00003320, #31S409).

## Conflict of interest

The authors declare that the research was conducted in the absence of any commercial or financial relationships that could be construed as a potential conflict of interest.

## Publisher’s note

All claims expressed in this article are solely those of the authors and do not necessarily represent those of their affiliated organizations, or those of the publisher, the editors and the reviewers. Any product that may be evaluated in this article, or claim that may be made by its manufacturer, is not guaranteed or endorsed by the publisher.

## Footnotes

^1^ http://www.worldclim.org/

^2^ https://imr.bio/

^3^ https://doi.org/10.5281/zenodo.6371857

## References

[ref1] Alsharif W. Saad M. M. Hirt H. (2020). Desert microbes for boosting sustainable agriculture in extreme environments. Front. Microbiol. 11:1666. doi: 10.3389/fmicb.2020.01666, PMID: 32793155 PMC7387410

[ref2] Amin A. Ahmed I. Habib N. Abbas S. Hasan F. Xiao M.. (2016). Microvirga pakistanensis sp. nov., a novel bacterium isolated from desert soil of Cholistan, Pakistan. Arch. Microbiol. 198, 933–939. doi: 10.1007/s00203-016-1251-3, PMID: 27290649

[ref3] Asmar S. Sassi M. Phelippeau M. Drancourt M. (2016). Inverse correlation between salt tolerance and host-adaptation in mycobacteria. BMC. Res. Notes 9:249. doi: 10.1186/s13104-016-2054-y, PMID: 27129386 PMC4850692

[ref4] Bakker M. G. Chaparro J. M. Manter D. K. Vivanco J. M. (2015). Impacts of bulk soil microbial community structure on rhizosphere microbiomes of *Zea mays*. Plant Soil 392, 115–126. doi: 10.1007/s11104-015-2446-0

[ref6] Blanchet F. G. Legendre P. Borcard D. (2008). Forward selection of explanatory variables. Ecology 89, 2623–2632. doi: 10.1890/07-0986.1, PMID: 18831183

[ref7] Callahan B. J. McMurdie P. J. Rosen M. J. Han A. W. Johnson A. J. A. Holmes S. P. (2016). DADA2: high-resolution sample inference from Illumina amplicon data. Nat. Methods 13, 581–583. doi: 10.1038/nmeth.3869, PMID: 27214047 PMC4927377

[ref8] Chen L. J. Li C. S. Feng Q. Wei Y. P. Zhao Y. Zhu M.. (2019). An integrative influence of saline water irrigation and fertilization on the structure of soil bacterial communities. J. Agric. Sci. 157, 693–700. doi: 10.1017/S002185962000012X

[ref9] Chen L. Li C. Feng Q. Wei Y. Zheng H. Zhao Y.. (2017). Shifts in soil microbial metabolic activities and community structures along a salinity gradient of irrigation water in a typical arid region of China. Sci. Total Environ. 598, 64–70. doi: 10.1016/j.scitotenv.2017.04.105, PMID: 28437772

[ref10] Cox T. L. Sly L. I. (1997). Phylogenetic relationships and uncertain taxonomy of Pedomicrobium species. Int. J. Syst. Bacteriol. 47, 377–380. doi: 10.1099/00207713-47-2-377, PMID: 9103624

[ref11] Dai T. Zhang Y. Tang Y. Bai Y. Tao Y. Huang B.. (2016). Identifying the key taxonomic categories that characterize microbial community diversity using full-scale classification: a case study of microbial communities in the sediments of Hangzhou Bay. FEMS Microbiol. Ecol. 92:fiw150. doi: 10.1093/femsec/fiw150, PMID: 27402713

[ref12] Dhariwal A. Chong J. Habib S. King I. L. Agellon L. B. Xia J. (2017). MicrobiomeAnalyst: a web-based tool for comprehensive statistical, visual and meta-analysis of microbiome data. Nucleic Acids Res. 45, W180–W188. doi: 10.1093/nar/gkx295, PMID: 28449106 PMC5570177

[ref13] Egamberdieva D. Renella G. Wirth S. Islam R. (2010). Secondary salinity effects on soil microbial biomass. Biol. Fertil. Soils 46, 445–449. doi: 10.1007/s00374-010-0452-1

[ref14] Futamata H. Sakai M. Ozawa H. Urashima Y. Sueguchi T. Matsuguchi T. (1998). Chemotactic response to amino acids of fluorescent pseudomonads isolated from spinach roots grown in soils with different salinity levels. Soil Sci. Plant Nutr. 44, 1–7. doi: 10.1080/00380768.1998.10414421

[ref15] Gschwend F. Hartmann M. Mayerhofer J. Hug A.-S. Enkerli J. Gubler A.. (2022). Site and land-use associations of soil bacteria and fungi define core and indicative taxa. FEMS Microbiol. Ecol. 97, 1–14. doi: 10.1093/femsec/fiab165, PMID: 34940884 PMC8752248

[ref16] Guo H. Ma L. Liang Y. Hou Z. Min W. (2020a). Response of ammonia-oxidizing Bacteria and Archaea to long-term saline water irrigation in alluvial grey desert soils. Sci. Rep. 10:489. doi: 10.1038/s41598-019-57402-x, PMID: 31949227 PMC6965641

[ref17] Guo H. Shi X. Ma L. Yang T. Min W. (2020b). Long-term irrigation with saline water decreases soil nutrients, diversity of bacterial communities, and cotton yields in a gray desert soil in China. Pol. J. Environ. Stud. 29, 4077–4088. doi: 10.15244/pjoes/120158

[ref18] Guo J. Zhou Y. Guo H. Min W. (2021). Saline and alkaline stresses alter soil properties and composition and structure of gene-based nitrifier and denitrifier communities in a calcareous desert soil. BMC Microbiol. 21:246. doi: 10.1186/s12866-021-02313-z, PMID: 34521348 PMC8442331

[ref19] Herlemann D. P. Labrenz M. Jürgens K. Bertilsson S. Waniek J. J. Andersson A. F. (2011). Transitions in bacterial communities along the 2000 km salinity gradient of the Baltic Sea. ISME J. 5, 1571–1579. doi: 10.1038/ismej.2011.41, PMID: 21472016 PMC3176514

[ref20] Hou Y. Zeng W. Hou M. Wang Z. Luo Y. Lei G.. (2021). Responses of the soil microbial community to salinity stress in maize fields. Biology (Basel). 10:1114. doi: 10.3390/biology10111114, PMID: 34827107 PMC8614889

[ref21] Ibekwe A. M. Ors S. Ferreira J. F. S. Liu X. Suarez D. L. (2017). Seasonal induced changes in spinach rhizosphere microbial community structure with varying salinity and drought. Sci. Total Environ. 579, 1485–1495. doi: 10.1016/j.scitotenv.2016.11.151, PMID: 27916300

[ref22] Karmakar J. Goswami S. Pramanik K. Maiti T. K. Kar R. K. Dey N. (2021). Growth promoting properties of Mycobacterium and Bacillus on rice plants under induced drought. Plant Sci. Today 8, 49–57. doi: 10.14719/pst.2021.8.1.965

[ref23] Khan Q. Kalbus E. Alshamsi D. M. Mohamed M. M. Liaqat M. U. (2019). Hydrochemical analysis of groundwater in remah and Al Khatim regions, United Arab Emirates. Hydrology 6:60. doi: 10.3390/hydrology6030060

[ref24] Khan M. A. Khan S. T. (2020). Microbial communities and their predictive functional profiles in the arid soil of Saudi Arabia. Soil 6, 513–521. doi: 10.5194/soil-6-513-2020

[ref25] Kristensen J. M. Singleton C. Clegg L.-A. Petriglieri F. Nielsen P. H. (2021). High diversity and functional potential of Undescribed “Acidobacteriota” in Danish wastewater treatment plants. Front. Microbiol. 12:643950. doi: 10.3389/fmicb.2021.643950, PMID: 33967982 PMC8100337

[ref26] Langille M. G. I. Zaneveld J. Caporaso J. G. McDonald D. Knights D. Reyes J. A.. (2013). Predictive functional profiling of microbial communities using 16S rRNA marker gene sequences. Nat. Biotechnol. 31, 814–821. doi: 10.1038/nbt.2676, PMID: 23975157 PMC3819121

[ref27] Li Y. Q. Chai Y. H. Wang X. S. Huang L. Y. Luo X. M. Qiu C.. (2021b). Bacterial community in saline farmland soil on the Tibetan plateau: responding to salinization while resisting extreme environments. BMC Microbiol. 21:119. doi: 10.1186/s12866-021-02190-6, PMID: 33874905 PMC8056723

[ref28] Li X. Wang A. Wan W. Luo X. Zheng L. He G.. (2021a). High salinity inhibits soil bacterial community mediating nitrogen cycling. Appl. Environ. Microbiol. 87:e0136621. doi: 10.1128/AEM.01366-21, PMID: 34406835 PMC8516042

[ref29] Mandic-Mulec I. Stefanic P. van Elsas J. D. (2015). Ecology of Bacillaceae. Microbiol. Spectr. 3, 1–24. doi: 10.1128/microbiolspec.TBS-0017-2013 26104706

[ref30] Martínez S. A. Dussán J. (2018). *Lysinibacillus sphaericus* plant growth promoter bacteria and lead phytoremediation enhancer with *Canavalia ensiformis*. Environ. Prog. Sustain. Energy 37, 276–282. doi: 10.1002/ep.12668

[ref31] Marvasi M. Visscher P. T. Casillas Martinez L. (2010). Exopolymeric substances (EPS) from *Bacillus subtilis*: polymers and genes encoding their synthesis. FEMS Microbiol. Lett. 313, 1–9. doi: 10.1111/j.1574-6968.2010.02085.x, PMID: 20735481

[ref32] Moroenyane I. Tripathi B. M. Dong K. Sherman C. Steinberger Y. Adams J. (2018). Bulk soil bacterial community mediated by plant community in Mediterranean ecosystem, Israel. Appl. Soil Ecol. 124, 104–109. doi: 10.1016/j.apsoil.2017.10.035

[ref33] Mukhtar S. Mehnaz S. Malik K. A. (2021). Comparative study of the rhizosphere and root endosphere microbiomes of Cholistan desert plants. Front. Microbiol. 12:618742. doi: 10.3389/fmicb.2021.618742, PMID: 33841349 PMC8032897

[ref34] Nan L. Guo Q. Cao S. Zhan Z. (2022). Diversity of bacterium communities in saline-alkali soil in arid regions of Northwest China. BMC Microbiol. 22:11. doi: 10.1186/s12866-021-02424-7, PMID: 34991470 PMC8734156

[ref35] Nelson D. W. Sommers L. E. (1996). “Total carbon, organic carbon, and organic matter,” in Methods of Soil Analysis. eds. Sparks D. Page A. Helmke P. Loeppert R. Soltanpour P. N. Tabatabai M. A.. (John Wiley & Sons, Ltd), 961–1010.

[ref36] O’Brien F. J. M. Almaraz M. Foster M. A. Hill A. F. Huber D. P. King E. K.. (2019). Soil salinity and pH drive soil bacterial community composition and diversity along a lateritic slope in the Avon river critical zone observatory, Western Australia. Front. Microbiol. 10:1486. doi: 10.3389/fmicb.2019.01486, PMID: 31312189 PMC6614384

[ref37] Oksanen J. Blanchet F. G. Friendly M. Kindt R. Legendre P. McGlinn D.. (2020). Vegan: community ecology package. Available at: https://cran.r-project.org/package=vegan (Accessed November 16, 2018).

[ref38] Qiao C. Duan Y. Zhang M. Hagemann M. Luo Q. Lu X. (2018). Effects of reduced and enhanced glycogen pools on salt-induced sucrose production in a sucrose-secreting strain of *Synechococcus elongatus* PCC 7942. Appl. Environ. Microbiol. 84, 1–11. doi: 10.1128/AEM.02023-17, PMID: 29101204 PMC5752869

[ref39] Quast C. Pruesse E. Yilmaz P. Gerken J. Schweer T. Yarza P.. (2012). The SILVA ribosomal RNA gene database project: improved data processing and web-based tools. Nucleic Acids Res. 41, D590–D596. doi: 10.1093/nar/gks1219, PMID: 23193283 PMC3531112

[ref40] Rath K. M. Fierer N. Murphy D. V. Rousk J. (2019). Linking bacterial community composition to soil salinity along environmental gradients. ISME J. 13, 836–846. doi: 10.1038/s41396-018-0313-8, PMID: 30446737 PMC6461869

[ref41] Rath K. M. Maheshwari A. Rousk J. (2017). The impact of salinity on the microbial response to drying and rewetting in soil. Soil Biol. Biochem. 108, 17–26. doi: 10.1016/j.soilbio.2017.01.018

[ref01] R Core Team (2020). R: A language and environment for statistical computing. Vienna, Austria. Available at: https://www.R-project.org/

[ref42] Ren M. Zhang Z. Wang X. Zhou Z. Chen D. Zeng H.. (2018). Diversity and contributions to nitrogen cycling and carbon fixation of soil salinity shaped microbial communities in Tarim basin. Front. Microbiol. 9:431. doi: 10.3389/fmicb.2018.00431, PMID: 29593680 PMC5855357

[ref43] Rengasamy P. (2018). Irrigation water quality and soil structural stability: A perspective with some new insights. Agronomy 8:72. doi: 10.3390/agronomy8050072

[ref44] Rognes T. Flouri T. Nichols B. Quince C. Mahé F. (2016). VSEARCH: a versatile open source tool for metagenomics. PeerJ 4:e2584. doi: 10.7717/peerj.2584, PMID: 27781170 PMC5075697

[ref45] Yu X. Jin Z. Wang H. (2021). Effect of saline water for drip irrigation on microbial diversity and on fertility of aeolian sandy soils. Diversity 13:379. doi: 10.3390/d13080379

[ref46] Yuan C. Feng S. Wang J. Huo Z. Ji Q. (2018). Effects of irrigation water salinity on soil salt content distribution, soil physical properties and water use efficiency of maize for seed production in arid Northwest China. Int. J. Agric. Biol. Eng. 11, 137–145. doi: 10.25165/j.ijabe.20181103.3146

[ref47] Zaitsev G. M. Tsitko I. V. Rainey F. A. Trotsenko Y. A. Uotila J. S. Stackebrandt E.. (1998). New aerobic ammonium-dependent obligately oxalotrophic bacteria: description of *Ammoniphilus oxalaticus* gen. Nov., sp. nov. and *Ammoniphilus oxalivorans* gen. Nov., sp. nov. Int. J. Syst. Bacteriol. 48, 151–163. doi: 10.1099/00207713-48-1-151, PMID: 9542085

[ref48] Zhang K. Shi Y. Cui X. Yue P. Li K. Liu X.. (2019). Salinity is a key determinant for soil microbial communities in a desert ecosystem. mSystems 4, e00225–e00218. doi: 10.1128/mSystems.00225-18, PMID: 30801023 PMC6372838

[ref49] Zhang W. Zhou G. Li Q. Liao N. Guo H. Min W.. (2016). Saline water irrigation stimulate N2O emission from a drip-irrigated cotton field. Acta Agric. Scand. Sect. B—Soil Plant Sci. 66, 141–152. doi: 10.1080/09064710.2015.1084038

[ref50] Zheng W. Xue D. Li X. Deng Y. Rui J. Feng K.. (2017). The responses and adaptations of microbial communities to salinity in farmland soils: a molecular ecological network analysis. Appl. Soil Ecol. 120, 239–246. doi: 10.1016/j.apsoil.2017.08.019

