# Supplementary material for: Irrigation water salinity structures the bacterial communities of date palm (Phoenix dactylifera)-associated bulk soil
Source: Front Plant Sci. 2022 Aug 4;13:944637. doi: 10.3389/fpls.2022.944637 (PMC9388049; doi:10.3389/fpls.2022.944637)
Supplement: Supplementary file 1 [file Data_Sheet_1.docx]

# **Supplementary Information for:**

Irrigation water salinity structures the bacterial communities of date palm (*Phoenix dactylifera*)-associated bulk soil

Dinesh Sanka Loganathachetti^1^, Fardous Alhashmi^1^, Subha Chandran^1^, Sunil Mundra^*, 1,2^

^1^Department of Biology, College of Science, United Arab Emirate University, Al Ain, United Arab Emirates

^2^Khalifa Center for Genetic Engineering and Biotechnology, United Arab Emirates University, P.O. Box. 15551, Al Ain, UAE

*** Correspondence:**

Sunil Mundra
[sunilmundra@uaeu.ac.ae](mailto:sunilmundra@uaeu.ac.ae)

Figures: One (S1)

Tables: Three (S1 to S3)

**Figure S1. Soil and water chemistry between irrigation water sources (non-saline water and saline groundwater irrigation).** The box plots of (a) water pH, (b) water electrical conductivity (EC in ds m^-1^), and (c) soil organic matter (OM%) under different irrigation water sources (non-saline vs saline groundwater irrigation). The P values of ANOVA followed by Tukey’s HSD post hoc test are given within each panel. The box spans the interquartile range (IQR; first quartile to the third) with the median indicated by a dark horizontal line and the whiskers indicating the 1.5×IQR. Data for each sample is also displayed with a strip chart.


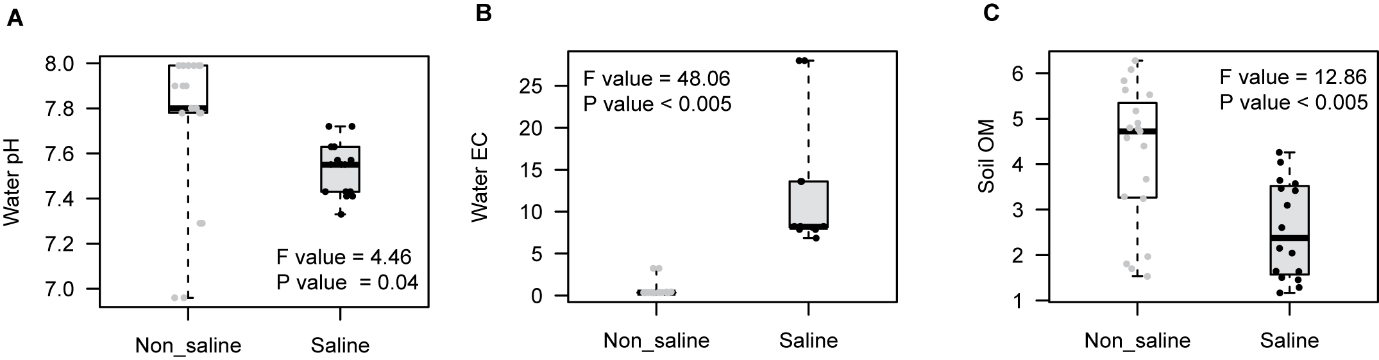


# **Table S1. The geo-climatic metadata of sampling sites.** The latitude, longitude, mean annual temperature, and mean annual precipitation data of sampling sites are represented based on the past 50 years of data from the worldclim database.

| **Location** | **Site name** | **Latitude** | **Longitude** | **Mean annual temperature (ºC)** | **Mean annual precipitation**  **(mm)** |
| --- | --- | --- | --- | --- | --- |
| Town_center | NS1 | 24°12'58.20"N | 55°45'9.80"E | 27.7 | 75 |
| Nahel_1 | NS2 | 24°54'29.05" N | 55°62'08.33"E | 25.3 | 109 |
| Nahel_2 | NS3 | 24°52'01.5" N | 55°65'89.06"E | 25.3 | 109 |
| Nahel_3 | NS4 | 24°53'51.95" N | 55°60'58.67" E | 25.3 | 109 |
| Al_rawda | NS5 | 24°06'03.9"N | 55°32'08.6"E | 27.8 | 75 |
| Seah_sharkiya | NS6 | 24°12'12.66"N | 55°48'53.40"E | 27.7 | 75 |
| Nabbagh | NS7 | 24°18'06.0"N | 55°43'08.9"E | 27.7 | 75 |
| Sarooj | S1 | 24°12'08.0"N | 55°47'18.1"E | 27.7 | 75 |
| Nahel_1 | S2 | 24°52'02.13" N | 55°64'43.09" E | 25.3 | 109 |
| Nahel_2 | S3 | 24°52'25.56" N | 55°65'45.12" E | 25.3 | 109 |
| Nahel_3 | S4 | 24°52'02.13" N | 55°64'43.09" E | 25.3 | 109 |
| Nahshala | S5 | 24°24'38.49" N | 55°23'53.55"E | 27.4 | 83 |
| Seah_salem_east | S6 | 24°20'44.2"N | 55°27'39.9"E | 27.4 | 83 |
| Seah_salem_west | S7 | 24°20'45.5"N | 55°26'24.4"E | 27.4 | 83 |

**Table S2. Taxonomic (phylum and order level) composition of the bacterial community in soil under non-saline water and saline groundwater irrigation.** Total % reads and % occurrences from the overall dataset and subsets are shown. Phyla with >1% of reads and orders with >0.5% of total reads are displayed. ^*^ Occurrence (%) was calculated from all 35 samples; ^#^ Occurrence (%) was calculated from all 19 non-saline samples; ^$^ Occurrence (%) was calculated from all 16 saline samples.

| **Taxonomy** | **Overall dataset** | | **Non-saline** | | **Saline** | |
| --- | --- | --- | --- | --- | --- | --- |
|  | **Read (%)** | **Occurrence (%)** | **Read (%)** | **Occurrence (%)^*^** | **Reads (%)** | **Occurrences (%)^#^** |
| Acidobacteriota | 9.24 | 100 | 9.10 | 100 | 9.41 | 100 |
| NA | 0.64 | 100 | 0.61 | 100 | 0.66 | 100 |
| Pyrinomonadales | 0.54 | 91.43 | 0.57 | 94.74 | 0.50 | 87.50 |
| Thermoanaerobaculales | 0.91 | 100 | 0.69 | 100 | 1.18 | 100 |
| Vicinamibacterales | 4.66 | 100 | 4.83 | 100 | 4.45 | 100 |
| Actinobacteriota | 23.73 | 100 | 24.34 | 100 | 23.01 | 100 |
| Actinomarinales | 6.56 | 100 | 6.31 | 100 | 6.86 | 100 |
| Corynebacteriales | 0.80 | 100 | 0.71 | 100 | 0.91 | 100 |
| Gaiellales | 2.79 | 100 | 3.03 | 100 | 2.51 | 100 |
| IMCC26256 | 0.64 | 100 | 0.67 | 100 | 0.60 | 100 |
| Micrococcales | 1.27 | 100 | 1.51 | 100 | 0.99 | 100 |
| Micromonosporales | 1.27 | 100 | 1.30 | 100 | 1.24 | 100 |
| Microtrichales | 2.16 | 100 | 2.23 | 100 | 2.08 | 100 |
| NA | 2.73 | 100 | 2.66 | 100 | 2.81 | 100 |
| Propionibacteriales | 1.15 | 100 | 1.39 | 100 | 0.86 | 100 |
| Pseudonocardiales | 0.61 | 100 | 0.63 | 100 | 0.58 | 100 |
| Solirubrobacterales | 1.47 | 100 | 1.60 | 100 | 1.31 | 100 |
| Streptomycetales | 0.65 | 100 | 0.66 | 100 | 0.63 | 100 |
| Chloroflexi | 10.63 | 100 | 11 | 100 | 10.19 | 100 |
| Ardenticatenales | 0.69 | 100 | 0.48 | 100 | 0.94 | 100 |
| Caldilineales | 0.74 | 97.14 | 0.59 | 100 | 0.91 | 93.75 |
| NA | 4.49 | 100 | 4.94 | 100 | 3.96 | 100 |
| S085 | 0.82 | 100 | 0.84 | 100 | 0.80 | 100 |
| SBR1031 | 0.99 | 100 | 0.93 | 100 | 1.05 | 100 |
| Thermomicrobiales | 1.71 | 100 | 1.87 | 100 | 1.52 | 100 |
| Firmicutes | 21.95 | 100 | 23.25 | 100 | 20.41 | 100 |
| Bacillales | 15.91 | 100 | 17.41 | 100 | 14.14 | 100 |
| Paenibacillales | 2.64 | 100 | 2.51 | 100 | 2.81 | 100 |
| Peptostreptococcales-Tissierellales | 0.84 | 97.14 | 1.14 | 100 | 0.49 | 93.75 |
| Thermoactinomycetales | 0.57 | 100 | 0.47 | 100 | 0.67 | 100 |
| Gemmatimonadota | 3.66 | 100 | 3.58 | 100 | 3.76 | 100 |
| Gemmatimonadales | 1.98 | 100 | 2.06 | 100 | 1.89 | 100 |
| NA | 1.61 | 100 | 1.44 | 100 | 1.82 | 100 |
| Methylomirabilota | 1.39 | 100 | 1.48 | 100 | 1.29 | 100 |
| Rokubacteriales | 1.39 | 100 | 1.48 | 100 | 1.29 | 100 |
| Myxococcota | 2.34 | 100 | 2.63 | 100 | 2 | 100 |
| Polyangiales | 0.87 | 100 | 0.97 | 100 | 0.75 | 100 |
| Planctomycetota | 1.87 | 100 | 1.74 | 100 | 2.02 | 100 |
| Pirellulales | 0.92 | 100 | 0.83 | 100 | 1.03 | 100 |
| Proteobacteria | 25.18 | 100 | 22.88 | 100 | 27.91 | 100 |
| Burkholderiales | 1.80 | 97.14 | 1.72 | 100 | 1.90 | 93.75 |
| Caulobacterales | 0.65 | 100 | 0.53 | 100 | 0.78 | 100 |
| CCD24 | 0.87 | 97.14 | 0.89 | 100 | 0.84 | 93.75 |
| NA | 1.97 | 100 | 1.58 | 100 | 2.43 | 100 |
| PLTA13 | 1.07 | 97.14 | 0.91 | 100 | 1.25 | 93.75 |
| Pseudomonadales | 0.73 | 97.14 | 0.37 | 100 | 1.17 | 93.75 |
| Rhizobiales | 10.88 | 100 | 10.83 | 100 | 10.93 | 100 |
| Rhodobacterales | 0.95 | 100 | 0.62 | 100 | 1.35 | 100 |
| Sphingomonadales | 0.55 | 100 | 0.50 | 100 | 0.61 | 100 |
| Steroidobacterales | 0.90 | 100 | 0.85 | 100 | 0.96 | 100 |
| Tistrellales | 2 | 100 | 1.82 | 100 | 2.21 | 100 |

**Table S3. The indicator operational taxonomic units (OTUs) of soil under non-saline water and saline groundwater irrigation.** IndVal >0.5 are listed in the table, indicating the degree of association between an OTU and site group. Statistical significance (P-value) was calculated after 999 times of permutation.

| **OTUs** | **Genus** | **IndVal** | **P value** | **Group** |
| --- | --- | --- | --- | --- |
| OTU_645 | *Solirubrobacter* | 0.668 | 0.0001 | Non–saline |
| OTU_1061 | *Sorangium* | 0.608 | 0.0003 | Non–saline |
| OTU_346 | *Geminicoccus* | 0.589 | 0.0001 | Non–saline |
| OTU_145 | *AKYG1722_unclassified* | 0.576 | 0.0002 | Non–saline |
| OTU_637 | *Lysobacter* | 0.522 | 0.0005 | Non–saline |
| OTU_576 | *67-14_unclassified* | 0.51 | 0.0018 | Non–saline |
| OTU_1282 | *Mycobacterium* | 0.536 | 0.0002 | Saline |
| OTU_33 | *Steroidobacter* | 0.506 | 0.0003 | Saline |
